# Supplementary material for: Impact of Malnutrition on Long-Term Mortality in Elderly Patients with Acute Myocardial Infarction
Source: Nutrients. 2019 Jan 22;11(2):224. doi: 10.3390/nu11020224 (PMC6412515; doi:10.3390/nu11020224)
Supplement: Supplementary file 1 [file nutrients-11-00224-s001.pdf]

Table S1. Baseline Characteristics based on MNA score

**Baseline Characteristics**	**MNA < 24 (*N*=88)**	**MNA ≥ 24 (*N*=86)**	***p*-value**
Age, years ± SD	74.50 ± 6.86	74.01 ± 7.33	0.651
Gender, male (*n*,%)	53 (60.2)	61 (70.9)	0.153
Weight, kg ± SD	71.70 ± 16.72	75.93 ± 10.45	0.047
BMI, kg/m^2 ± SD	27.03 ± 6.72	28.09 ± 4.08	0.207
Haemoglobin, mg/dl ± SD	12.95 ± 1.77	13.15 ± 2.0	0.484
Glycemia, mg/dl, ± SD	129.92 ± 49.24	141.62 ± 57.36	0.151
Total Cholesterol, mg/dl ± SD	195.75 ± 69.62	207.51 ± 64.40	0.250
Albumine mg/dl ± SD	3.65 ± 0.73	3.81 ± 0.49	0.080
GFR ml/kg/m2 ± SD	73.04 ± 32.07	71.73 ± 24.50	0.763
Hypertension, (*n*, %)	64 (72.7)	63 (73.2)	0.537
COPD, (*n*, %)	21 (23.7)	18 (20.9)	0.717
DM, (*n*, %)	32 (36.3)	30 (34.9)	0.875
STEMI, (*n*, %)	48 (54.5)	44 (51.2)	0.761
Death, (*n*, %)	32 (36.4)	11(12.8)	<0.001

Continuous variables are expressed as mean ± SD, binary data as percentage. BMI, body mass index; DM, diabetes mellitus; COPD, chronic obstructive pulmonary disease; GFR, glomerular filtration rate; STEMI, ST-elevated myocardial infarction.
